# Supplementary material for: Volcanic crystals as time capsules of eruption history
Source: Nat Commun. 2018 Jan 23;9:326. doi: 10.1038/s41467-017-02274-w (PMC5780494; doi:10.1038/s41467-017-02274-w)
Supplement: Supplementary file 1 — Supplementary Information [file 41467_2017_2274_MOESM1_ESM.pdf]

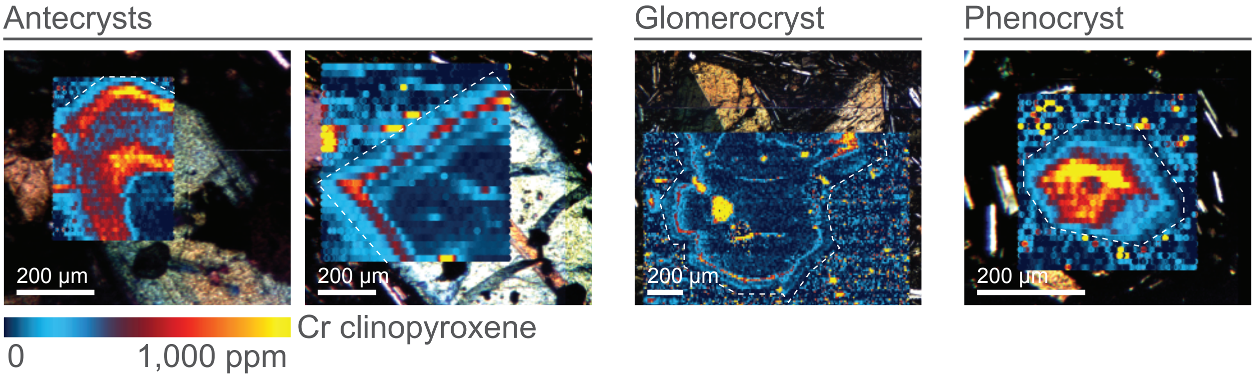

Supplementary Figure 1. Chromium maps of clinopyroxene crystals from the 1974 eruption at Mt. Etna. Chromium-poor, often resorbed antecryst cores crystallised prior to the intrusion of primitive magma. Chromium-rich zones crystallised upon arrival of mafic magma, followed by Cr-poor zones that we relate to decompression and degassing upon final magma ascent and eruption. Note Cr-rich zones overgrowing complex glomerocrysts assembled before recharge. New phenocrysts formed after intrusion have Cr-rich cores and Cr-poor rims.

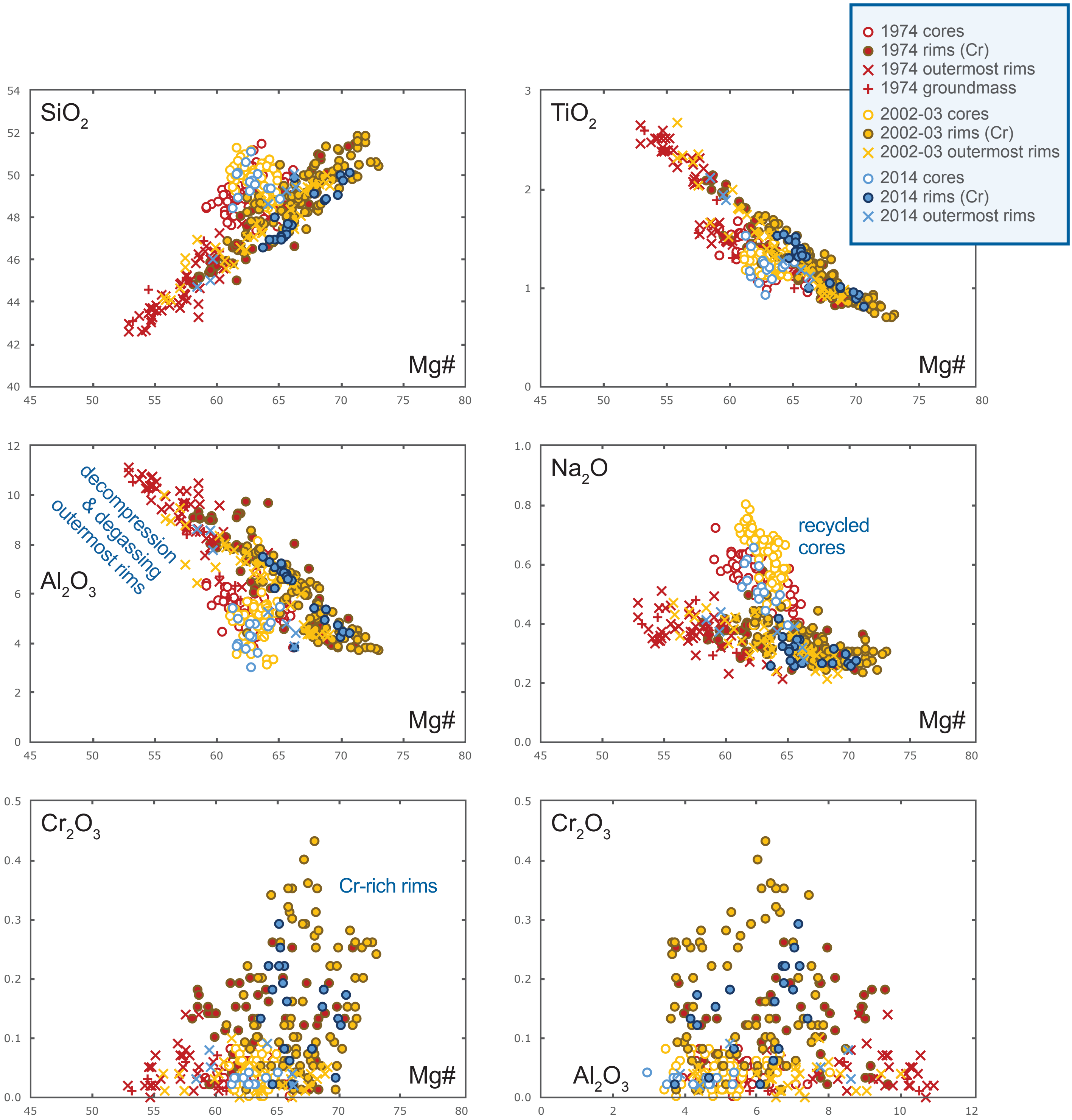

Supplementary Figure 2. Major element variations in clinopyroxene populations. Crystal cores typically have high Na concentrations, suggesting they were recycled from deeper mushes[44]. Cr-rich rims, Cr-poor outermost rims and groundmass microcrysts define a linear trend that suggests fractionation from the same (mafic intrusion) magma. Outermost rims show Al-Ti-enrichments coupled with Si-Mg#-depletions, indicating crystallisation related to magma decompression and higher undercooling[41]. Note the primitive nature of the intruding magma, which crystallised high Cr-Mg# rims. There is no correlation between Cr and Al, supporting the interpretation that Cr-uptake is not related to local enrichment of slow diffusing elements at the liquid boundary layer surrounding rapidly growing crystals. Mg# = 100 MgO / (MgO + total iron as FeO).

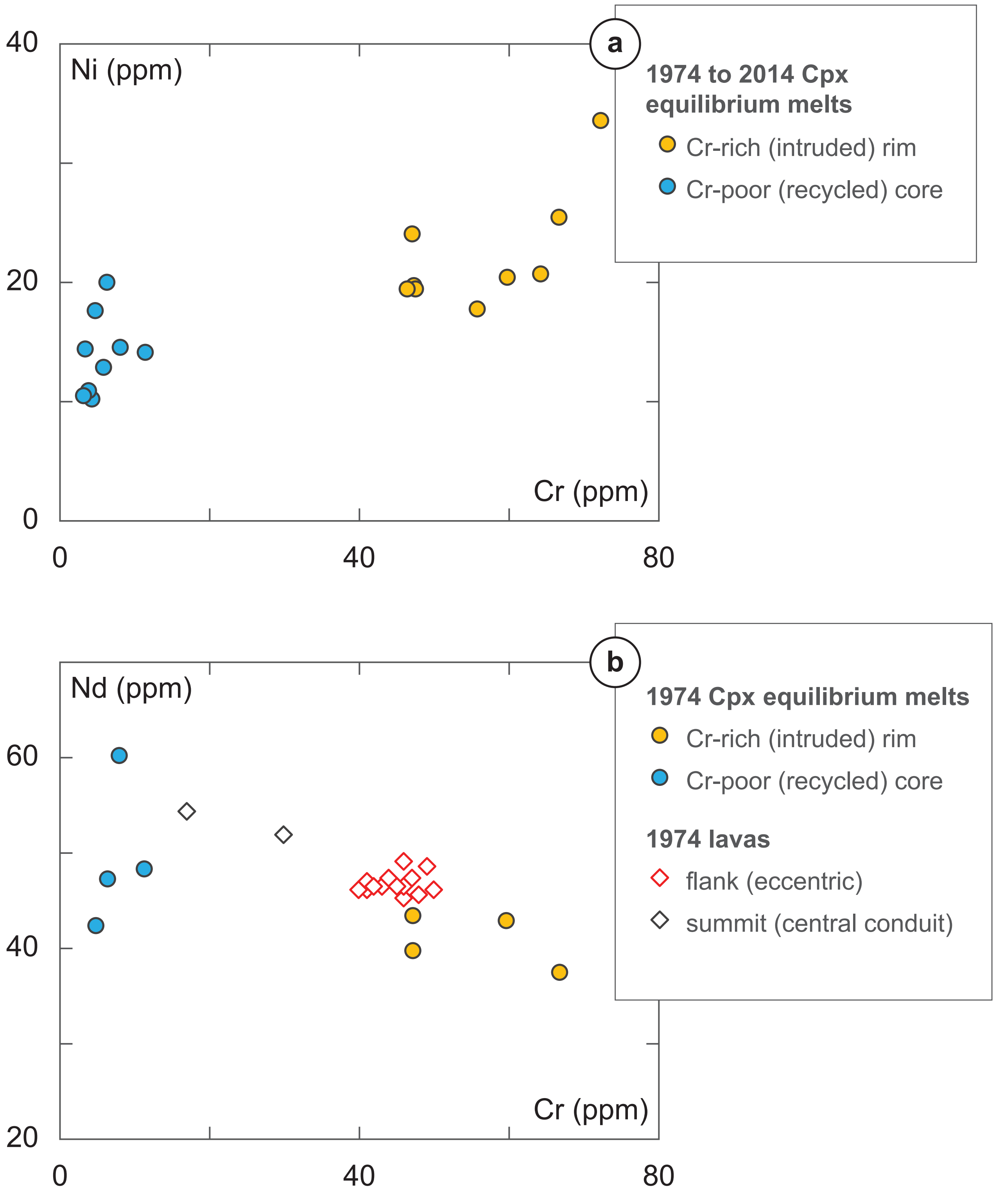

Supplementary Figure 3. Melts in equilibrium with Cr-rich and Cr-poor clinopyroxene. a) Positive correlations between Cr and other transition metals (e.g., Ni) in equilibrium melts confirm recharge with batches of more primitive magma. Data extracted from clinopyroxene in eruptions spanning the period 1974-2014. The compositional gap is a reflection of inefficient hybridisation, potentially enlarged by extracting end-member compositions only. b) Nd (incompatible) vs. Cr (compatible) concentrations of melts in equilibrium with 1974 Cr-rich and Cr-poor clinopyroxene, compared to bulk lava compositions from 1974 eccentric and central conduit eruptions[23]. Lavas plot as hybrids between the two end-members: those fed from eccentric dykes plot close to the Cr-rich end-member whereas those fed from the central conduits plot close to the Cr-poor end-member.

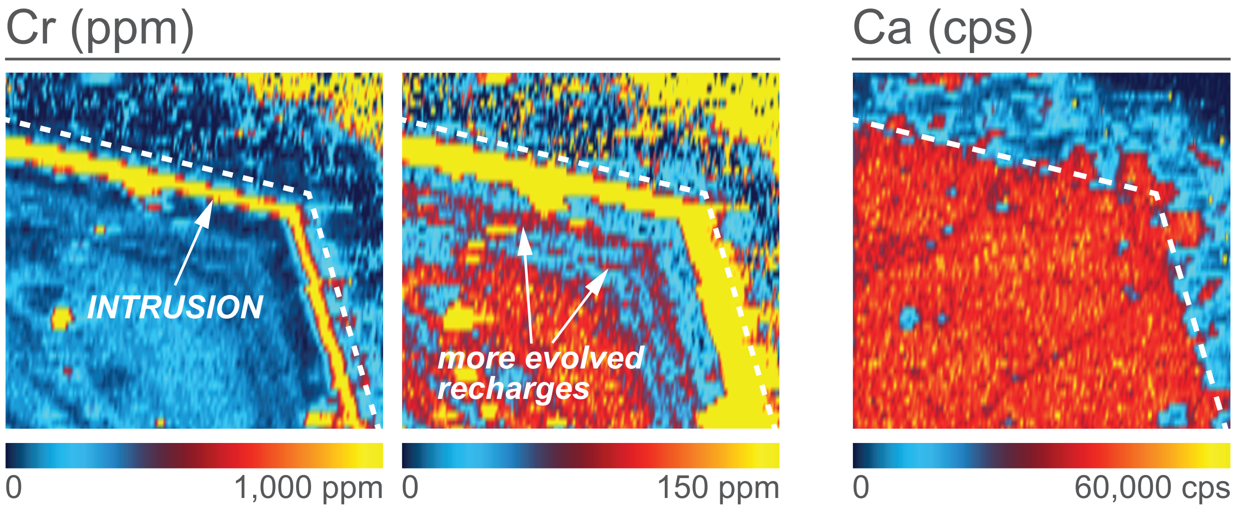

Supplementary Figure 4. Chromium maps of the clinopyroxene antecryst in Fig. 1 using different limits on the linear scale. Note that the recycled core is oscillatory zoned in Cr but at levels one order of magnitude lower than the Cr-rich rim crystallised upon magma intrusion. Oscillatory zoning in the core therefore indicates older, more evolved recharge events. The raw counts per second map of Ca, used as internal standard for all mapping experiments, illustrates homogeneity regardless of zonation in other elements.

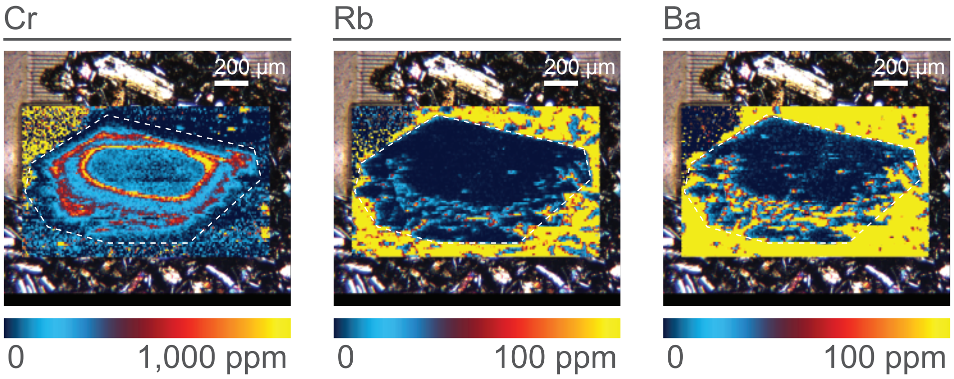

Supplementary Figure 5. Alkaline element maps of clinopyroxene. Chromium enrichments in clinopyroxene are not related to changes in alkaline elements such as Rb and Ba. Note however that these elements are strongly incompatible in clinopyroxene[39] and concentrations are close to detection limits.

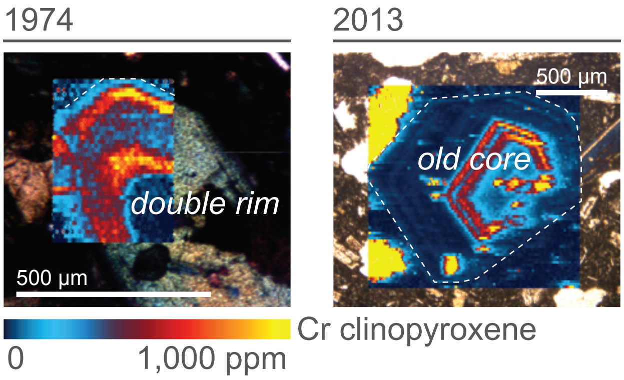

Supplementary Figure 6. Examples of crystals with intermediate Cr-enrichments. The crystal from the 1974 eccentric eruption has a double rim that could be related to double recharge or swirling of magma and migration of crystals upon intrusion[5,47]. The crystal from the 2013 central conduit-fed eruption has a double enrichment in the mantle area probably related to previous intrusion/s that did not reach the surface, as the higher crystallinity in the central conduits might delay magma mobilisation. Intermediate Cr-enrichments could potentially be much older than eruption, kept in cold storage[11] in the plumbing system. Thus for intrusion statistics (Fig. 3; Supplementary Table 4), we disregarded intermediate Cr-enrichments and only considered Cr-rich antecryst rims and phenocryst cores.

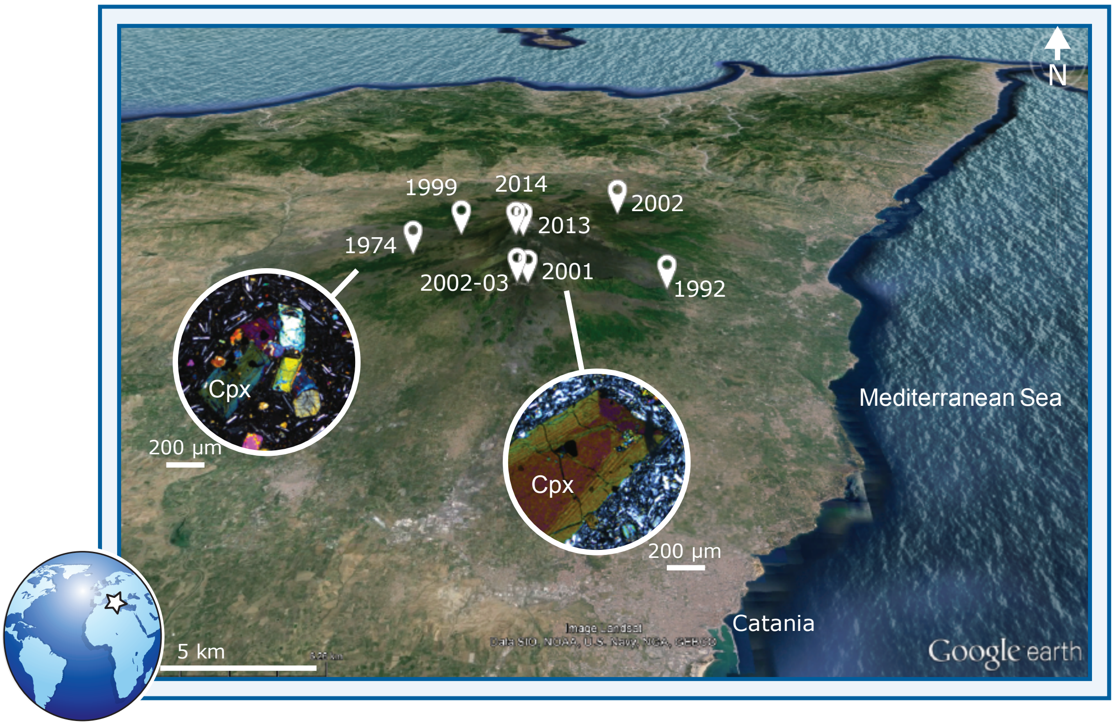

Supplementary Figure 7. Location of sampling sites at Mt Etna. The stratovolcano is located on the eastern coast of Sicily (Italy). White pins mark the location of the sampled lava flows and their eruption age on a Google Earth image. The circles show example photomicrographs (transmitted light, crossed polars) of the 1974 and 2001 eccentric eruptions.

Supplementary Table 1. Eruption and location of studied samples at Mt. Etna.

Sample	Eruption	Type	Coordinates (UTM 33S)	
15RC004	1974	Eccentric	493513	4177191
15RC008	1974	Eccentric	493493	4177507
15RC013	1974	Eccentric	493465	4177724
15RC014	1974	Eccentric	493451	4177682
15RC015	1974	Eccentric	493436	4177558
15RC017	1974	Eccentric	493347	4177339
ETNAB1	1974	Eccentric	493421	4177339
15TU109	1992	Central conduit	508095	4173782
15TU111	1999	Central conduit	495749	4178881
15RC022	2001 lower vents	Eccentric	500255	4172462
15TU108	2002 north flank	Central conduit	505386	4183281
15RC023	2002-03 south flank	Eccentric	499998	4172521
15TU106	2002-03 south flank	Eccentric	500336	4175410
15TU105	2013	Central conduit	499563	4176352
15TU104	2014	Central conduit	499467	4176377

Supplementary Table 2. Instrument parameters used for LA-ICPMS experiments.

Laser parameters	Fluence (J/cm2)	2.5-3.5
	Spot size (µm)*	24x24 / 20x20 / 14 / 12x12
	Stage translation speed (µm/s)*	36 / 30 / 10 / 18
	Repetition rate (Hz)*	10 / 10 / 20 / 10
	Overlap between rasters in maps (µm)	1
	Background between rasters (s)	30-40
Gas flows	He (ml/min)	700-800
	Ar (ml/min)	650-750
	N2 (ml/min)	6-9
ICP-MS parameters	Analytes	43Ca, 45Sc, 52Cr, 60Ni, 71Ga, (85Rb), 88Sr, 90Zr, 93Nb, (137Ba), 139La, 146Nd
	Individual dwell times (ms)	5-20
	Duty cycle (ms)	100-140
Total experiment time (min)		30-180

* Spatial resolution adjusted with a combination of spot size, translation speed and repetition rate (ref. 37). Four sets of parameters, separated by slashes.

Supplementary Table 3. Extracted clinopyroxene compositions from Cr-rich and Cr-poor regions in selected clinopyroxene crystal maps from Mt. Etna 1974-2014 eruptions, and calculated compositions of liquids in equilibrium using partition coefficients from ref. 39.

	Cr-rich											Cr-poor										
Eruption	1974	1974	1974	1974	1999	2001	2002-03	2002-03	2014			1974	1974	1974	1974	1999	2001	2002-03	2002-03	2014		
Sample	15RC004	15RC008	15RC017	15RC017	15TU111	15RC022	15RC023	15TU106	15TU104			15RC004	15RC008	15RC017	15RC017	15TU111	15RC022	15RC023	15TU106	15TU104		
Type	Antecryst	Antecryst	Glomerocrys	Phenocryst	Antecryst	Antecryst	Antecryst	Antecryst	Antecryst	***Average***	*St dev*	Antecryst	Antecryst	Glomerocrys	Phenocryst	Antecryst	Antecryst	Antecryst	Antecryst	Antecryst	***Average***	*St dev*
Clinopyroxene																						
Sc (ppm)	174	144	176	207	125	125	142	132	140	***152***	*28*	138	112	121	179	109	113	103	117	113	***123***	*23*
Cr (ppm)	804	803	1135	1017	951	1228	808	1094	791	***959***	*167*	196	110	83	139	75	61	67	102	56	***99***	*45*
Ni (ppm)	69	84	89	71	62	117	68	73	68	***78***	*17*	50	70	62	51	36	50	38	45	37	***49***	*12*
Ga (ppm)	11	16	15	14	9	10	10	9	9	***11***	*3*	12	14	15	16	12	15	12	10	11	***13***	*2*
Sr (ppm)	121	123	116	121	109	89	108	105	114	***112***	*11*	123	131	121	127	150	118	133	118	125	***127***	*10*
Zr (ppm)	114	98	92	143	65	43	92	61	64	***86***	*31*	137	92	103	199	140	163	129	85	119	***130***	*36*
Nb (ppm)	1	1	1	1	1	0	1	0	1	***1***	*0*	3	1	1	3	3	2	2	1	1	***2***	*1*
La (ppm)	10	9	8	9	5	3	8	6	5	***7***	*2*	15	11	10	14	20	20	17	10	13	***14***	*4*
Nd (ppm)	30	27	26	29	18	13	23	18	16	***22***	*6*	33	32	29	41	45	49	46	28	36	***38***	*8*
Melt in equilibrium																						
Sc (ppm)	26	22	27	31	19	19	22	20	21	***23***	*4*	21	17	18	27	17	17	16	18	17	***19***	*4*
Cr (ppm)	47	47	67	60	56	72	48	64	47	***56***	*10*	12	6	5	8	4	4	4	6	3	***6***	*3*
Ni (ppm)	20	24	26	20	18	34	19	21	19	***22***	*5*	14	20	18	14	10	14	11	13	10	***14***	*3*
Ga (ppm)																						
Sr (ppm)	861	879	828	866	779	633	771	748	815	***798***	*77*	881	934	862	908	1071	843	951	841	890	***909***	*72*
Zr (ppm)	227	196	183	287	130	86	184	121	127	***171***	*62*	274	184	206	398	281	327	257	171	238	***260***	*72*
Nb (ppm)	25	25	19	34	12	7	16	11	23	***19***	*9*	81	33	30	73	79	52	35	21	24	***48***	*24*
La (ppm)	54	50	46	51	28	19	42	32	30	***39***	*12*	85	62	56	78	110	109	95	55	74	***81***	*21*
Nd (ppm)	43	40	38	43	26	18	35	26	24	***32***	*9*	48	47	42	60	67	72	67	42	52	***55***	*11*

Suplementary Table 4. Statistical analysis of clinopyroxene crystal populations per eruption. Eccentric eruptions are marked in bold.

	**1974**	1992	1999	**2001**	2002	**2002-03**	2013	2014	*Total*	*Average*	***Total Eccentric***	***Average Eccentric***
All crystals analysed												
n	**15**	38	34	**33**	19	**62**	42	44	*287*		***110***	
% Macrocrysts >1.5 mm	**13**	5	0	**21**	16	**19**	5	9		*11*		***18***
% Mesocrysts 0.5-1.5 mm	**67**	50	38	**52**	63	**56**	57	64		*56*		***58***
% microcrysts <0.5 mm	**20**	45	62	**27**	21	**24**	38	27		*33*		***24***
% as single crystals	**60**	11	12	**67**	68	**84**	19	23		*43*		***70***
% as glomerocrysts	**40**	89	88	**33**	32	**16**	81	77		*57*		***30***
% Cr-rich cores	**20**	0	3	**21**	5	**16**	5	5		*9*		***19***
% Cr-rich mantles	**27**	11	24	**36**	0	**2**	14	0		*14*		***22***
% Cr-rich rims	**73**	13	3	**76**	0	**52**	10	43		*34*		***67***
% intruded crystals*	**93**	13	6	**82**	5	**68**	14	48		*41*		***81***
Antecrysts with intruded rims												
n	**11**	5	1	**25**	0	**32**	4	19	*97*		***68***	
Cr-rich rims												
Thickness (µm)	**32**	23	37	**43**	-	**83**	47	134		*57*		***53***
St dev (µm)	**16**	10	-	**24**	-	**56**	25	66		*33*		***32***
% of crystal growth	**7**	10	9	**9**	-	**16**	11	31		*13*		***11***
Time crystallisation (days)**	**4**	3	4	**5**	-	**10**	5	16		*7*		***6***
St dev (days)	**2**	1	-	**3**	-	**7**	3	8		*4*		***4***
Cr-poor outermost rims												
Thickness (µm)	**65**	14	60	**48**	-	**75**	44	60		*52*		***63***
St dev (µm)	**34**	9	-	**46**	-	**56**	15	78		*40*		***45***
% of crystal growth	**13**	5	14	**9**	-	**15**	12	13		*11*		***12***
Time crystallisation (days)**	**8**	2	7	**6**	-	**9**	5	7		*6*		***7***
St dev (days)	**4**	1	-	**5**	-	**7**	2	9		*5*		***5***

*Intruded crystals group only antecrysts with Cr-rich rims and phenocrysts with Cr-rich cores

**Timescales of crystallisation calculated according to a growth rate of 10-8 cm/s (refs. 26, 54, 55). For Cr-poor overgrowths crystallised upon magma ascent, timescales are maxima
