# Supplementary material for: Volcanic crystals as time capsules of eruption history
Source: Nat Commun. 2018 Jan 23;9:326. doi: 10.1038/s41467-017-02274-w (PMC5780494; doi:10.1038/s41467-017-02274-w)
Supplement: Supplementary file 3 — Description of Additional Supplementary Files [file 41467_2017_2274_MOESM3_ESM.pdf]

Description of Additional Supplementary Files

File Name: Supplementary Data 1
Description: Database of clinopyroxene crystals analysed per eruption using LA-ICPMS maps or transects. For each crystal, the table includes analytical conditions, crystal size, occurrence as single crystal or glomerocryst, presence of Crrich cores, mantles and rims, their thickness and their distance to the crystal rim, as well as growth timescales.

File Name: Supplementary Data 2
Description: Database of in-situ FE-SEM-EDS spot analyses on clinopyroxene. For each analysis, the table includes information on the crystal zone analysed (core, Cr-rich rim, Cr-poor outermost rim or groundmass microcryst) as well as the calculation of the structural formula and the thermobarometry results.
